# Endotracheal Tube Cuff Pressures in Patients Intubated Prior to Helicopter EMS Transport

**DOI:** 10.5811/westjem.2016.8.30639

**Published:** 2016-09-13

**Authors:** Joseph Tennyson, Tucker Ford-Webb, Stacy Weisberg, Donald LeBlanc

**Affiliations:** * University of Massachusetts Medical School, Department of Emergency Medicine, Division of Emergency Medical Services, Worcester, Massachusetts; † Lahey Hospital & Medical Center, Emergency Department, Burlington, Massachusetts; ‡ UMass Memorial LifeFlight, Emergency Medical Services, Worcester, Massachusetts

## Abstract

**Introduction:**

Endotracheal intubation is a common intervention in critical care patients undergoing helicopter emergency medical services (HEMS) transportation. Measurement of endotracheal tube (ETT) cuff pressures is not common practice in patients referred to our service. Animal studies have demonstrated an association between the pressure of the ETT cuff on the tracheal mucosa and decreased blood flow leading to mucosal ischemia and scarring. Cuff pressures greater than 30 cmH_2_O impede mucosal capillary blood flow. Multiple prior studies have recommended 30 cmH_2_O as the maximum safe cuff inflation pressure. This study sought to evaluate the inflation pressures in ETT cuffs of patients presenting to HEMS.

**Methods:**

We enrolled a convenience sample of patients presenting to UMass Memorial LifeFlight who were intubated by the sending facility or emergency medical services (EMS) agency. Flight crews measured the ETT cuff pressures using a commercially available device. Those patients intubated by the flight crew were excluded from this analysis as the cuff was inflated with the manometer to a standardized pressure. Crews logged the results on a research form, and we analyzed the data using Microsoft Excel and an online statistical analysis tool.

**Results:**

We analyzed data for 55 patients. There was a mean age of 57 years (range 18–90). The mean ETT cuff pressure was 70 (95% CI= [61–80]) cmH_2_O. The mean lies 40 cmH_2_O above the maximum accepted value of 30 cmH_2_O (p<0.0001). Eighty-four percent (84%) of patients encountered had pressures above the recommended maximum. The most frequently recorded pressure was >120 cmH_2_O, the maximum pressure on the analog gauge.

**Conclusion:**

Patients presenting to HEMS after intubation by the referral agency (EMS or hospital) have ETT cuffs inflated to pressures that are, on average, more than double the recommended maximum. These patients are at risk for tracheal mucosal injury and scarring from decreased mucosal capillary blood flow. Hospital and EMS providers should use ETT cuff manometry to ensure that they inflate ETT cuffs to safe pressures.

## INTRODUCTION

Endotracheal intubation is a common intervention in critical care patients undergoing helicopter emergency medical services (HEMS) transportation. A standard adult endotracheal tube (ETT) is secured at its distal end in the trachea using an inflatable cuff. This cuff serves to minimize aspiration risk and provides a seal to allow for delivery of a positive pressure gradient. The pressure in an ETT cuff must be high enough to occlude the lumen of the trachea in order to serve these primary functions.

Excess pressure, however, may increase the risk of damage to the tracheal mucosa.^1^–^3^ ETT cuff pressures (ETTCP) that exceed the capillary perfusion pressure of the mucosa upon which the cuff is pressing may prevent the flow of blood through those capillaries and lead to mucosal ischemia.^2^,^3^ Animal and human studies have demonstrated that ETTCP in excess of 30 cmH_2_O may cause decreased blood flow to the tracheal mucosa in as little as 25 minutes.^1^–^3^ While guidelines for inflation pressures exist, 4 available equipment to measure cuff pressure is not routinely used in all settings, and even experienced operators are prone to over-inflation.^5^–^7^

We hypothesized that in patients intubated by referral EMS agencies or referral hospitals, the initial cuff pressure measured by the HEMS crew would be within the accepted safe range.

Reduction in tracheal blood flow as a consequence of higher-than-recommended ETTCP has been associated with ischemic lesions to the trachea.^8^ Identifying the frequency at which patients are presented for transfer with ETTCP higher-than-recommended safe values will allow modification of practice.

## METHODS

### Study Design and Setting

We performed a prospective cohort study of patients intubated by referring agencies, both hospitals and EMS agencies, who presented for critical care transport by UMass Memorial Life Flight. The study was approved by the University of Massachusetts Medical School Institutional Review Board.

### Selection of Participants

This study was performed at UMass Memorial Life Flight, a critical care transport service based in Worcester, MA, between 2013 and 2014. Patients who were intubated by referring agencies (hospitals or EMS agencies) and transported by helicopter were consecutively included in the study. We excluded patients if they were prisoners at the time of transfer, or if they had been intubated with non-cuffed ETTs.

### Methods and Measurements

**Figure 1:**
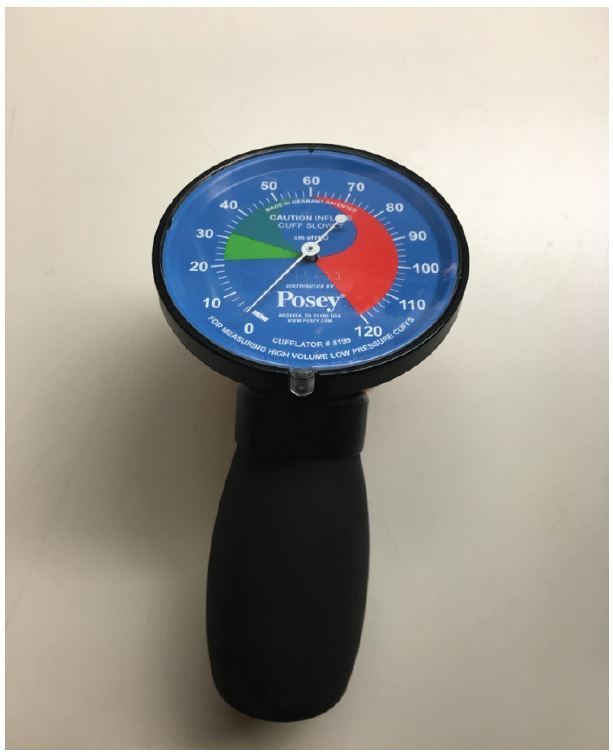
Posey Cufflator™ endotracheal tube inflator and manometer.

In all patients intubated prior to initial LifeFlight contact, a baseline ETT cuff pressure reading was obtained at the time of initial assessment. If the pressure was in excess of 25mmH_2_O, it was lowered to that pressure. Pressure measurements, inflation, and deflation of the ETT cuffs were performed using the Posey Cufflator™ endotracheal tube inflator and manometer (Posey Company, 5635 Peck Road, Arcadia, California 91006-0020 USA), a commercially available device. The maximum measurement on this device is >120 cmH_2_O (see Figure 1).

### Data Collection

Data were collected by critical care paramedics and nurses and entered at the time of measurement into a data collection form created for the purpose of the study. These data were then transcribed to a computer database for analysis.

### Outcome Measures

The primary endpoint of the study was the ETTCP of ETTs placed by referral agencies.

### Data Analysis

This study is an observational cohort of a series collected to analyze the change in pressure of ETT cuffs with altitudinal changes in flight. This paper represents a pre-planned subgroup analysis of the initial ETTCP of patients intubated prior to UMass Memorial Life Flight arrival. The original study was planned for 110 patients based on a pre-hoc power calculation. We analyzed the data analyzed at midpoint (55 patients) and found them to be significant for this cohort. The data was entered into and analyzed using Microsoft Excel 2013 (Microsoft Corporation, Redmond, WA) and a Web-based statistical analysis tool for the one-sided T test. For the purposes of analysis of the data, we treated manometer readings at the maximum on this analog manometer (>120 cm H_2_O) as equal to 120 cmH_2_O.

## RESULTS

**Figure 2:**
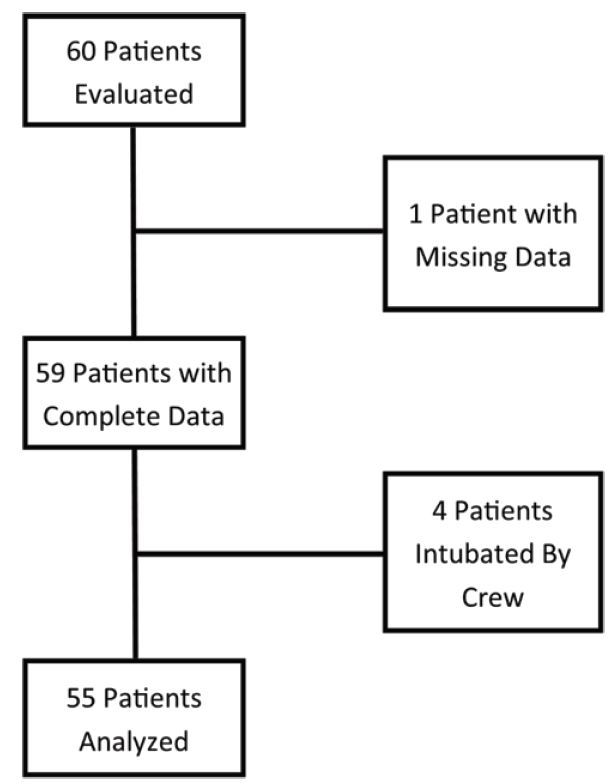
Flow chart of study patient selection and reasons for exclusion from analysis.

At the time data analysis was begun, 60 records had been entered into the database. One record was excluded for incomplete data (missing the initial cuff pressure). We excluded four additional records as the patients were not intubated prior to the Life Flight crew’s arrival and were intubated by the crew. The remaining 55 patients were analyzed (see Figure 2).

The table lists the characteristics of the analyzed cases. The mean age of patients was 57, ranging from 18 to 90. Male patients predominated by a small margin. More cases were related to medical conditions as opposed to traumatic conditions. The most common ETT size was 7.5 with sizes ranging from 6.0 to 8.5.

**Figure 3:**
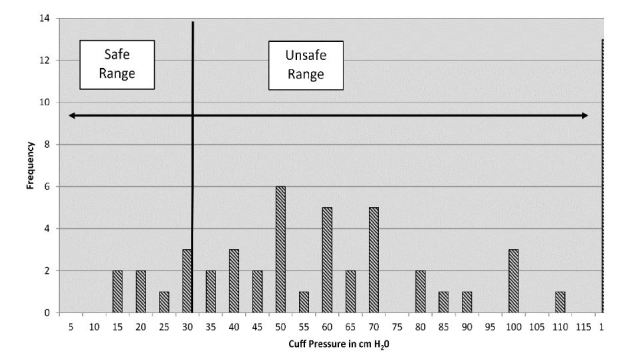
Distribution of initial endotracheal tube cuff pressures.

Initial ETTCP ranged from 15 cmH_2_O to >120 cm cmH_2_O. The mean pressure measurement was 70 cmH_2_O, 40 cmH_2_O higher than the accepted maximum safe value of 30 cmH_2_O (p<0.0001, 95% CI for the difference= [31–50]). The mode was >120cmH2O. Of the measurements, 8 (14.55%) were below the accepted maximum safe value of 30 cmH_2_O, 47 (85.45%) above that value. Figure 3 shows the distribution of results.

## DISCUSSION

The vast majority of endotracheal tubes transported by our critical care HEMS crew had a dangerous level of cuff over-inflation. Less than 15% of the measurements found pressures within acceptable ranges and the most common value was at the upper limit of the manometer’s range. Pressures such as this have been shown in animal studies to cause tissue ischemia to the tracheal mucosa.^2^,^3^

Evidence for the harm of over-inflation of ETT cuffs is not limited to animal studies. A 1984 study by Seegobin found blanching of tracheal mucosa on tracheoscopy in patients whose ETTCP exceeded 40 cm H_2_O.^1^ This blanching suggests decreased blood flow and ischemia to those regions. A 2013 paper by Touat et al used tracheoscopy on newly extubated patients to evaluate the degree of injury with a tracheal ischemia score. They found that ETTCP > 30 cm H_2_O was associated with an elevated tracheal ischemia score.^8^ This demonstrates that the issue persists despite the introduction of modern high-volume, low-pressure cuffs.

Less severe complications related to over-inflation of ETT cuffs include hoarseness, sore throat and hemoptysis.^8^ More severe complications include post-intubation stridor,^9^ tracheal stenosis^10^ and even reports of tracheal rupture.^11^,^12^ One study by Kastanos demonstrated a 10% rate of development of tracheal stenosis and that this demonstrated a statistically significant association with elevated ETTCP.^10^

The prevalence of over-inflated ETTCP has been reported several times and yet persists. The reports have covered clinical environments including the prehospital environment,^6^,^7^,^13^ the emergency department (ED),^13^ the peri-operative environment,^14^,^15^ and the intensive care unit (ICU).^16^,^17^

Many clinicians rely on pilot-balloon estimation of cuff pressures. The inaccuracy of this technique has been demonstrated many times.^9^,^14^,^18^,^19^ One study evaluated the accuracy of this method by certified nurse anesthetists and anesthesiologists as well as students. They found that fewer than one-third of the cuffs were inflated to an appropriate range. Further, they failed to demonstrate a difference in the accuracy of cuff inflation when stratified by provider experience.^14^

Techniques for using various-sized syringes as pressure-relief valves have been published over the years.^20^,^21^ This technique is analogous to the pilot-balloon technique in that a syringe is left connected to the pilot balloon, allowing the air pressure in the cuff to move the syringe plunger when it is too high. While the early reports favored this technique, a more recent report has found it lacking.^22^

In this study each of the abnormal pressures was normalized prior to flight. Had the pressures not been normalized the risk of tracheal injury might have been even higher. Several papers have demonstrated that ETTCP is affected by altitude changes^23^ when patients are transported by aeromedical transport modes.^24^–^27^ This analysis of patients presenting for HEMS transport demonstrated that the majority began with pressure outside the safe range. Our data suggest uncorrected pressures could lead to severe worsening pressures as the patient is brought to altitude, increasing the risk of severe complications.

## LIMITATIONS

This dataset is limited by possible confounding variables that were not collected by the data collection forms. It is possible that identification of whether the intubation was performed by hospital staff or field EMS personnel may have identified a tendency toward over-inflation by one of those groups. Additionally, for those cases intubated in a hospital setting, delineating whether they were done in the ED, ICU, or operating room, may have also allowed for more stratification of the data. Finally, the question of who specifically inflated the cuff, be they physician, nurse, or respiratory therapist, may also have elucidated some associations that could potentially have suggested further research.

The fact that these data were collected from a single HEMS system may tend to limit the degree to which they can be generalized. Possibly offsetting this limitation is the fact that the subjects included in the study originated from multiple EMS systems and multiple referral hospitals across a five-state area, providing a greater cross-section than may be inferred from the single HEMS service.

## CONCLUSION

In conclusion, we present additional evidence that current standard practice in EMS agencies and referral hospitals in our HEMS system leads to frequently elevated ETTCP. These pressures place the patient at risk for complications from the ETT. Clinicians should move to routine measurement of ETTCP in all intubated patients.

## Figures and Tables

**Table:** Characteristics of subjects in study analyzing endotracheal tube (ETT) cuff pressures in patients arriving to the emergency department via helicopter emergency medical services.

Characteristics	Result
Age (years), mean (95% CI)	57 (51–62)
Minimum age	18
Maximum age	90
Gender, n (%)	
Male	35 (64)
Female	20 (36)
Nature of case, n (%)	
Trauma	10 (18)
Medical	45 (81)
ETT size	
Mode	7.5
Minimum ETT size	6.0
Maximum ETT size	8.5

*CI,* confidence interval
